# Case Report: Ischemic bowel necrosis following intra-aortic balloon pump support in a patient with cardiogenic shock

**DOI:** 10.3389/fcvm.2026.1845966

**Published:** 2026-09-22

**Authors:** XingZheng Luo, ChaoFeng Li, QuanQiu Ye, XiMin Lin, ShuQiang Liu, FengZheng Zhu, Ming Fang

**Affiliations:** 1 Department of Critical Care Medicine, Xiaolan Clinical Institute of Shantou University Medical College, Zhongshan, China; 2 Department of Critical Care Medicine, Xiaolan People's Hospital of Zhongshan (The Fifth People's Hospital of Zhongshan), Zhongshan, China; 3 Department of Cardiovascular Medicine, Xiaolan People's Hospital of Zhongshan (The Fifth People's Hospital of Zhongshan), Zhongshan, China; 4 Department of Gastrointestinal Surgery, Xiaolan People's Hospital of Zhongshan (The Fifth People's Hospital of Zhongshan), Zhongshan, China; 5 Department of Medical Imaging, Xiaolan People's Hospital of Zhongshan (The Fifth People's Hospital of Zhongshan), Zhongshan, China

**Keywords:** balloon-aortic length mismatch, cardiogenic shock, intra-aortic balloon pump, ischemic bowel necrosis, mesenteric ischemia

## Abstract

**Background:**

The intra-aortic balloon pump (IABP) is a widely used mechanical circulatory support device for patients with acute myocardial infarction complicated by cardiogenic shock. Although generally safe, IABP use may impair visceral organ perfusion and lead to rare but severe complications, including ischemic bowel necrosis.

**Case presentation:**

A 59-year-old man was admitted after experiencing a cardiac arrest caused by an extensive acute anterior myocardial infarction. Following successful cardiopulmonary resuscitation, emergency coronary angiography revealed total occlusion of the mid-segment of the left anterior descending artery. Primary percutaneous coronary intervention was performed with implantation of a drug-eluting stent and IABP support, achieving a thrombolysis in myocardial infarction grade 3 flow. On the fourth postoperative day, the patient developed progressive abdominal distension, absent bowel sounds, and refractory septic shock. Contrast-enhanced computed tomography revealed small bowel dilation with air-fluid levels, hepatic portal venous gas, and pneumatosis intestinalis, findings suggestive of ischemic bowel necrosis. Further evaluation indicated that the distal end of the intra-aortic balloon was positioned below the origin of the superior mesenteric artery, suggesting that a mismatch between the aortic anatomy and balloon length contributed to mesenteric hypoperfusion in the setting of cardiogenic shock and high-dose vasopressor therapy. The IABP counterpulsation ratio was adjusted to 1:2, and broad-spectrum antibiotics were administered. Despite these interventions, the patient's condition continued to deteriorate, with persistent high fever and circulatory collapse. The family ultimately decided to discontinue treatment due to unsustainable vital signs and a poor prognosis.

**Conclusion:**

This case highlights a rare and catastrophic complication following coronary stent implantation and IABP therapy in a patient with cardiogenic shock. Bowel ischemia in this setting is rarely attributable to a single mechanism: systemic hypoperfusion after cardiac arrest, IABP-related reduction in mesenteric blood flow due to balloon–aortic length mismatch, vasopressor-induced splanchnic vasoconstriction, and ischemia-reperfusion injury likely acted in concert. Clinicians should remain highly vigilant for early signs of ischemic bowel necrosis in patients receiving IABP support, particularly those requiring high doses of vasoactive agents, and should be aware that serum lactate trends may not reliably reflect splanchnic perfusion. Early recognition through serial abdominal examination and timely imaging is essential.

## Introduction

The intra-aortic balloon pump (IABP) is a critical mechanical circulatory support device extensively utilized in the management of critically ill patients with cardiogenic shock, acute myocardial infarction, or cardiac dysfunction following cardiac surgery (1). Despite advances in IABP technology, potential complications warrant careful consideration. Among these, secondary ischemic bowel necrosis is recognized as a rare yet exceedingly dangerous complication (2, 3). The development of this complication can significantly worsen the patient's condition and complicate treatment, and it is strongly associated with high mortality and poor prognosis. This article presents a case of ischemic bowel necrosis following IABP therapy for acute myocardial infarction. It reviews the relevant literature on its incidence, pathogenesis, diagnostic approaches, therapeutic strategies, and prognosis. The aim is to provide a reference for clinicians and to enhance awareness and response capabilities regarding this serious complication.

## Case presentation

A 59-year-old man, measuring 163 cm in height and weighing 59 kg, with an unremarkable medical history, was transferred to our hospital due to “chest pain lasting one day and sudden loss of consciousness lasting two hours.” The patient experienced chest discomfort one day before admission, but specific details were unavailable, and no medical attention was sought at that time. Two hours before admission, at 16:45 on December 19, 2025, the patient abruptly lost consciousness and collapsed. Emergency medical services arrived at 16:53 and assessed the patient in respiratory and cardiac arrest. Immediate interventions included cardiopulmonary resuscitation, intravenous administration of epinephrine, cardioversion, endotracheal intubation, and ventilator-assisted breathing. By 17:13, the patient had regained a ventricular rhythm at 98 beats per minute and had palpable femoral pulses. Resuscitation measures also included the administration of lidocaine, sodium bicarbonate, amiodarone, atropine, and dopamine. Sinus rhythm was restored at 18:14. Electrocardiography revealed ST-segment elevation in leads I, aVL, and V1–V5, with reciprocal depression in the inferior leads, indicating an extensive anterior myocardial infarction. Cardiac biomarker levels were notably high: Troponin I at 20.68 ng/mL (normal range: 0–0.3), CK-MB at 100 ng/mL (normal range: 0–5), and N-terminal pro-brain natriuretic peptide at 697.21 pg/mL (normal range: 0–300). Based on these findings, the patient was diagnosed with acute myocardial infarction, cardiogenic shock, and cardiac arrest. The patient was transferred to our hospital through the Chest Pain Center Alliance and arrived at 18:45 on December 19, 2025. On arrival, the patient was in a deep coma, with pupils that were round, equal in size, 2.0 mm in diameter, and unresponsive to light. The heart rate was 105 beats per minute, and blood pressure was 103/63 mmHg while receiving norepinephrine at 0.235 μg·kg^–1^·min^–1^. The patient was taken directly to the catheterization laboratory, skipping the emergency department and hospital ward. A brief timeline of the patient's clinical course is shown in Table 1.

**Table 1:** Clinical timeline of the patient's course, from cardiac arrest to withdrawal of care (per CARE case-report guidelines).

Time	Clinical event	Key findings and interventions
Day 0, 16:45	Out-of-hospital cardiac arrest	CPR, epinephrine, cardioversion, intubation; ROSC 17:13; sinus rhythm 18:14
Day 0, 18:45	Arrival via Chest Pain Center	Deep coma (GCS 3 T); extensive anterior STEMI; cardiogenic shock; norepinephrine 0.235 μg·kg^–1^·min^–1^
Day 0, 19:16	Emergency PCI + IABP insertion	Mid-LAD occlusion; 3.0 × 15 mm DES; TIMI 3 flow; IABP (IAB-S840C, 40 cc) via right femoral artery, 1:1 counterpulsation
Day 0, 19:55	ICU admission	Mechanical ventilation; targeted temperature management (35.0–36.0 °C); chest radiograph confirms IABP position (Figure 1)
Day 1	Postoperative day 1	Comatose; norepinephrine 0.142–0.508 and dopamine 6.5–11.3 μg·kg^–1^·min^–1^; lactate 3.4 mmol/L; LVEF 32%; APACHE-II 27
Day 2	Postoperative day 2	Gastric retention after enteral nutrition; mild abdominal distension; reduced bowel sounds
Day 3	Postoperative day 3	Coffee-ground gastric aspirate, occult blood positive; abdominal distension; bladder pressure 16 mmHg; lactate 3.2 mmol/L; abdominal CT deferred
Day 4	Diagnosis of ischemic bowel necrosis	Tense distended abdomen, absent bowel sounds; CTA: pneumatosis intestinalis, hepatic portal venous gas, balloon tip 32.3 mm distal to SMA ostium (Figure 2); IABP ratio reduced to 1:2; meropenem + vancomycin; laparotomy declined (irreversible brain injury); lactate 2.4 mmol/L
Day 4, 23:45	Withdrawal of care	Refractory septic shock (norepinephrine up to 1.725 μg·kg^–1^·min^–1^); family elected to withdraw life-sustaining treatment; patient discharged and died

Emergency coronary angiography revealed a normal left main coronary artery, 60%–70% stenosis in the proximal left anterior descending artery, and total occlusion of the mid-segment with a thrombolysis in myocardial infarction (TIMI) flow grade of 0. The left circumflex artery was patent, while the dominant right coronary artery exhibited 50%–70% stenosis in the proximal to mid-segment, with a TIMI flow grade of 3. An IABP catheter (model IAB-S840C, Teleflex Arrow International; 8 Fr shaft, 40 cc balloon volume, 260 mm balloon membrane length, and 15 mm inflated diameter, suitable for patients with a height of 162–182 cm) was inserted through the right femoral artery. With IABP support providing 1:1 assistance, a guidewire was successfully navigated through the lesion to the distal left anterior descending artery at 19:16. Following pre-dilation using a 2.5 × 20 mm balloon, a 3.0 × 15 mm drug-eluting stent was deployed, achieving a TIMI grade 3 flow. The door-to-wire time was recorded at 31 min. At 19:55 on December 19, 2025, the patient was transferred to the intensive care unit for ongoing treatment.

Upon admission to the intensive care unit (ICU), the patient was evaluated and found to be in a comatose state, with a Glasgow Coma Scale score of 3 T (E1VTM2). The pupils were bilaterally round and equal, measuring 2.0 mm in diameter, and were unresponsive to light. The patient remained on endotracheal intubation with ventilator-assisted breathing. Norepinephrine was administered at 0.140 μg·kg^–1^·min^–1^ to support circulatory function. Vital signs included a temperature of 36.2 ℃, a pulse rate of 67 beats per minute, a respiratory rate of 15 breaths per minute, a blood pressure of 103/57 mmHg, and an oxygen saturation (SpO2) of 98%. Cardiac rhythm was regular, although heart sounds were reduced. Bilateral basal lung rales were auscultated, and the extremities were cool. A chest x-ray confirmed the correct positioning of the IABP tip (Figure 1). Initial management in the ICU comprised mechanical ventilation, IABP support with 1:1 assistance, administration of vasoactive agents (norepinephrine at 0.042–0.055 μg·kg^–1^·min^–1^ and dopamine at 6.5–9.0 μg·kg^–1^·min^–1^), targeted temperature management (maintaining a temperature range of 35.0–36.0 ℃), dual antiplatelet therapy with aspirin and ticagrelor, and statin treatment.

**Figure 1:**
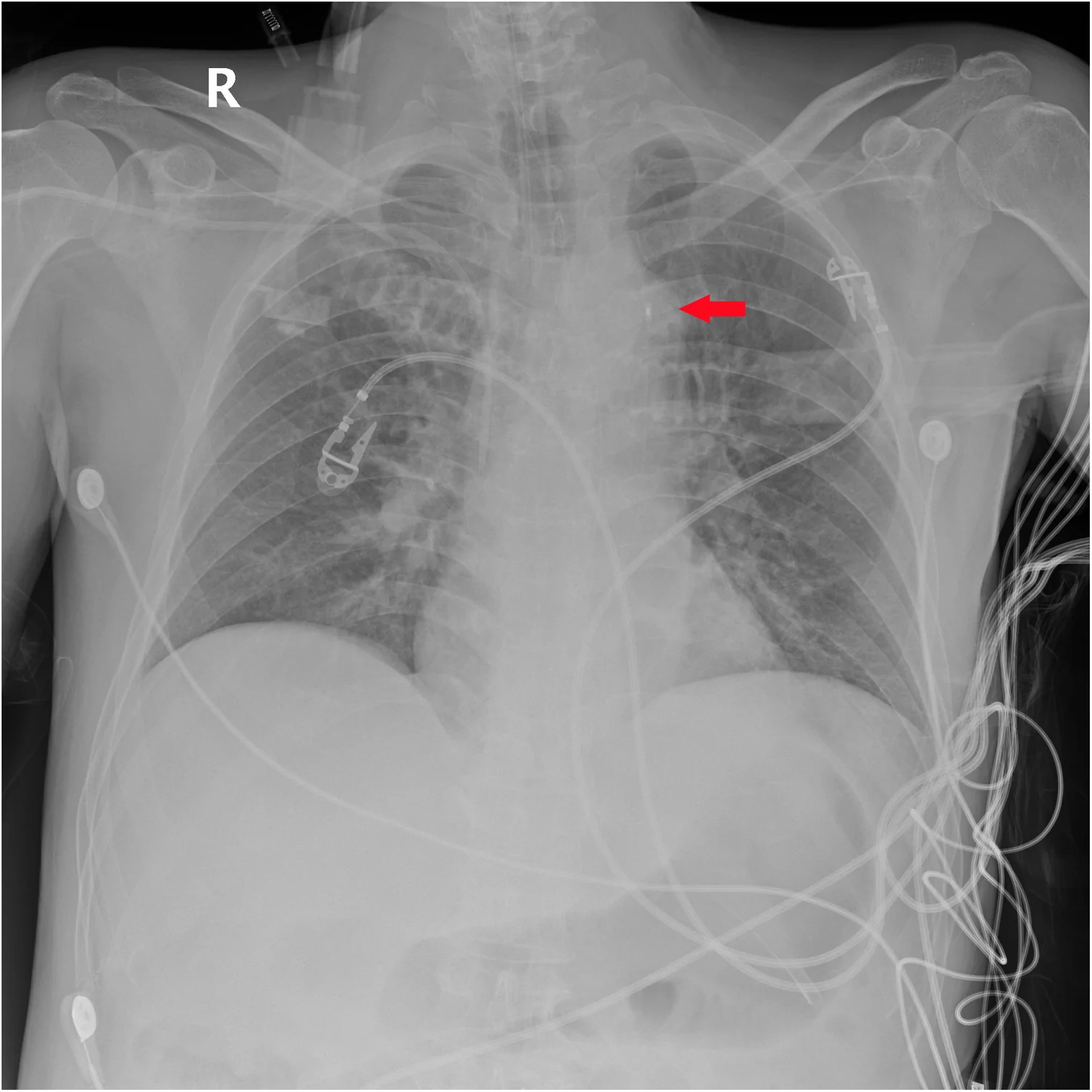
Chest radiograph obtained on the day of IABP insertion (day 0). The IABP marker was located at the second intercostal space, 26.7 mm from the thoracic aortic arch, indicating appropriate proximal positioning by conventional radiographic criteria. Note that this measurement uses the aortic arch as its anatomic reference on plain radiography and is distinct from the contrast-enhanced CT measurement on day 4, which localized the distal balloon tip 32.3 mm distal to the superior mesenteric artery ostium (Figure 2-D); the two values are complementary but not directly comparable.

On the first postoperative day (December 20, 2025), the patient remained in a comatose state with a Glasgow Coma Scale score of 3 T (E1VTM2). The pupils were bilaterally round and equal, measuring 2.0 mm in diameter, and were unresponsive to light. Intermittent limb twitching was noted. The patient was maintained on continuous sedation, analgesia, and targeted temperature management. To sustain blood pressure, norepinephrine was administered at a rate of 0.142–0.508 μg·kg^–1^·min^–1^ and dopamine at 6.5–11.3 μg·kg^–1^·min^–1^, with IABP support set at a 1:1 ratio. Physical examination revealed a temperature of 36.2 ℃, a pulse rate of 95 beats/min, a respiratory rate of 15 breaths/min, a blood pressure of 114/47 mmHg, and an SpO2 of 99%. Laboratory results indicated a white blood cell count of 21.93 × 10^9^/L with 83.6% neutrophils, a hemoglobin level of 158 g/L, a hematocrit of 0.45, and a platelet count of 212 × 10^9^/L. Cardiac biomarkers were elevated, with CK-MB > 600.00 μg/L, myoglobin at 1940.22 μg/L, high-sensitivity troponin T at 11,720 pg/mL, and N-terminal pro-brain natriuretic peptide at 2,658.8 pg/mL. Liver and renal function tests revealed a blood urea nitrogen level of 7.56 mmol/L, serum creatinine of 90.1 μmol/L, aspartate aminotransferase of 588.0 U/L, and alanine aminotransferase of 214.9 U/L. Arterial blood gas analysis revealed the following values: pH of 7.38, PaO2 of 152 mmHg, PaCO2 of 37 mmHg, lactate of 3.4 mmol/L, base excess of −2.7 mmol/L, and a PaO2/FiO2 ratio of 434 mmHg. Neuron-specific enolase was measured at 44.5 ng/mL, and procalcitonin at 1.760 ng/mL. Echocardiography indicated a left ventricular end-diastolic diameter of 53 mm, a left ventricular end-systolic diameter of 45 mm, and a left ventricular ejection fraction of 32%. The patient's APACHE-II score was 27, with an R value of 0.4081, and a SOFA score of 11.

On postoperative day 2 (December 21, 2025), the patient was receiving a continuous infusion of norepinephrine at 0.145–0.290 μg·kg^–1^·min^–1^ and dopamine at 5.8–7.7 μg·kg^–1^·min^–1^ for hemodynamic support, supplemented by IABP assistance at a 1:1 ratio. Gastric retention was observed after the initiation of enteral nutrition, and physical examination demonstrated mild abdominal distension with reduced bowel sounds.

On postoperative day 3 (December 22, 2025), the patient remained in a comatose state, with pupils that were bilaterally round, equal in size at 2.0 mm in diameter, and were unresponsive to light. Norepinephrine was administered at 0.193–0.386 μg·kg^–1^·min^–1^, while dopamine infusion remained at 5.8–7.7 μg·kg^–1^·min^–1^, with ongoing IABP support at a 1:1 ratio. The patient's heart rate and blood pressure remained stable. Gastric tube drainage indicated the presence of coffee-ground gastric contents, and an occult blood test returned positive results. Physical examination revealed abdominal distension and reduced bowel sounds. The measured bladder pressure was 16 mmHg. Laboratory analysis demonstrated a white blood cell count of 12.35 × 10^9^/L with 85.1% neutrophils, hemoglobin concentration of 157 g/L, hematocrit level of 0.45, and platelet count of 89 × 10^9^/L. Cardiac biomarkers indicated CK-MB levels at 24.45 μg/L and high-sensitivity troponin T at 4,594.00 pg/mL. Liver and renal function tests revealed blood urea nitrogen at 10.88 mmol/L, serum creatinine at 113.4 μmol/L, aspartate aminotransferase at 588.0 U/L, and alanine aminotransferase at 214.9 U/L. Arterial blood gas analysis demonstrated a pH of 7.40, PaO2 of 151 mmHg, PaCO2 of 31 mmHg, lactate at 3.2 mmol/L, base excess of −4.1 mmol/L, and a PaO2/FiO2 ratio of 412 mmHg. Neuron-specific enolase was measured at 2,462 ng/mL, and procalcitonin at 7.160 ng/mL. Ticagrelor therapy was discontinued, while aspirin antiplatelet therapy was continued. The clinical assessment suggested that the abdominal distension and diminished bowel sounds were attributable to analgesic and sedative medications and to the application of a cooling blanket; consequently, an abdominal computed tomography (CT) examination was not conducted.

On postoperative day 4 (December 23, 2025), the patient remained comatose, with bilaterally round, equal pupils 2.0 mm in diameter and absent light reflexes. The abdomen was observed to be distended, with a tense abdominal wall and the absence of bowel sounds. Laboratory investigations revealed a white blood cell count of 10.13 × 10^9^/L, with neutrophils comprising 86.0%, a hemoglobin level of 140 g/L, a hematocrit of 0.45, and a platelet count of 72 × 10^9^/L. Cardiac biomarkers indicated CK-MB at 18.94 μg/L, high-sensitivity troponin T at 6,038.00 pg/mL, and N-terminal pro-brain natriuretic peptide at 10,187.8 pg/mL. Renal function tests demonstrated blood urea nitrogen at 16.23 mmol/L and serum creatinine at 181.8 μmol/L. Arterial blood gas analysis demonstrated a pH of 7.34, PaO2 of 118 mmHg, PaCO2 of 41 mmHg, lactate at 2.4 mmol/L, base excess of −3.5 mmol/L, and a PaO2/FiO2 ratio of 295 mmHg. Inflammatory markers included C-reactive protein level of 390.1 mg/L and procalcitonin at 8.970 ng/mL. Echocardiographic assessment revealed a left ventricular end-diastolic diameter of 46 mm, a left ventricular end-systolic diameter of 29 mm, and a left ventricular ejection fraction of 67%. CT of the head and chest, along with contrast-enhanced abdominal CT, revealed the following findings: (1) Diffuse cerebral edema; (2) bilateral lower lobe consolidation consistent with aspiration pneumonia; (3) significant dilation of the small bowel with air-fluid levels, as illustrated in Figure 2C; (4) hepatic portal venous gas in the left medial segment (segment V), as illustrated in Figure 2A (white arrow), and pneumatosis intestinalis of the small bowel, as depicted in Figures 2B,C (white arrows); (5) the IABP balloon extending across the origins of the celiac trunk, superior mesenteric artery (SMA), and bilateral renal arteries, with its distal end positioned 32.3 mm distal to the SMA origin, as indicated in Figure 2D (red arrow). The minimum diameter of the descending aorta within the IABP-covered segment—its narrowest point, at the level of the renal artery origins (L1 vertebral body)—was 18.5 mm, and the inflated balloon diameter (15 mm) was assessed at the same level, corresponding to a balloon-to-aortic diameter ratio of 0.81. Based on clinical and imaging assessments, a diagnosis of intestinal ischemia with necrosis was established. Meropenem and vancomycin were administered as part of the anti-infective therapy regimen, and the IABP assistance ratio was adjusted to 1:2. A consultation with the gastrointestinal surgery team was obtained. Although ischemic bowel necrosis was considered, laparotomy was not recommended because of severe irreversible brain injury. Subsequently, the patient developed a significant fever, with body temperature rising to 39.2 ℃, accompanied by tachycardia with a heart rate of 130 beats per minute. Additionally, the patient's blood pressure required progressively higher doses of vasoactive agents, specifically norepinephrine at 1.725 μg·kg^–1^·min^–1^ and dopamine at 12.5 μg·kg^–1^·min^–1^, to maintain stability. Despite these interventions, the patient's condition continued to deteriorate. Due to multiple organ failure and an extremely poor prognosis, the patient's family declined further aggressive treatment and provided informed consent for the withdrawal of medical interventions. The patient was discharged at 23:45 on December 23, 2025.

**Figure 2:**
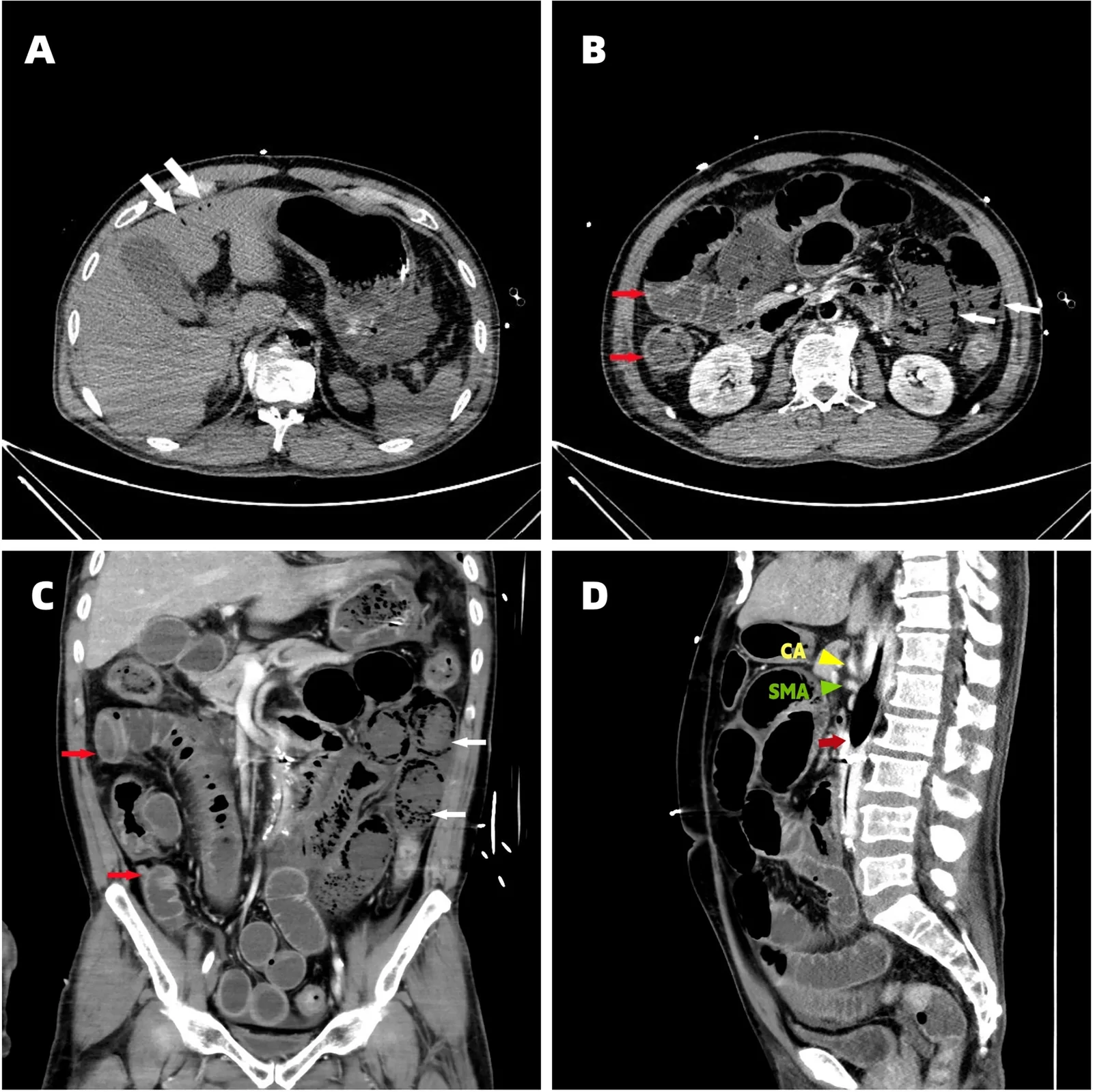
Abdominal computed tomography angiography obtained on day 4 following IABP insertion. Panel **(A)**, white arrows identify subcapsular hepatic portal venous gas. Panels **(B and C)** reveal the non-enhancement of the left small bowel wall, with a mean CT value of 21 HU, and the presence of intramural gas (indicated by white arrows), contrasting with the right small bowel wall, which has a mean CT value of 70 HU (indicated by red arrows). This is accompanied by notable small bowel dilation and fluid accumulation. Panel **(D)** (sagittal reconstruction) depicts the IABP balloon extending across the ostia of the celiac artery (CA, yellow arrowhead) and the superior mesenteric artery (SMA, green arrowhead); the balloon tip (indicated by a red arrow) is located 32.3 mm distal to the SMA ostium.

The final diagnoses included the following: (1) Acute extensive anterior myocardial infarction with cardiogenic shock (Killip Class IV); (2) post-cardiac arrest syndrome; (3) hypoxic-ischemic encephalopathy; (4) ischemic bowel necrosis with paralytic ileus; (5) intra-abdominal infection; (6) septic shock; (7) acute kidney injury, stage 2; (8) acute hepatic injury; (9) multiple organ dysfunction syndrome; (10) stress ulcer; (11) thrombocytopenia.

## Discussion

The reported incidence of IABP-induced ischemic bowel necrosis varies considerably across studies and clinical reports (4–6). This variability is largely due to differences in patient populations, study designs, diagnostic criteria, and the duration of IABP use. Although the absolute incidence is relatively low, IABP is often used in critically ill and high-risk patients, and the occurrence of severe complications can significantly affect clinical outcomes. Therefore, a thorough understanding of the epidemiological characteristics, pathogenesis, and diagnostic approaches of this condition is essential for developing effective prevention and treatment strategies.

Although generally described as rare, clinically important mesenteric ischemia is probably under-recognized. During perioperative IABP use for coronary artery bypass grafting, the incidence of mesenteric arterial ischemia reached 14.9% (4). In a Papworth Hospital autopsy series of 11,202 cardiac surgery patients, 42.3% of the 52 patients who developed fatal acute mesenteric ischemia had received IABP therapy, versus 2.4% of matched controls, and IABP use was a significant risk factor for fatal mesenteric ischemia (7)—suggesting that some patients die before a diagnosis can be made. Moreover, CT data from the Leipzig University Heart Center showed compromise of at least one abdominal visceral artery in 96.8% of IABP patients, including superior mesenteric artery (SMA) compression in 87.3% (6). Although not all flow impairment becomes clinically manifest, these figures underscore the pervasive influence of IABP on visceral circulation.

IABP-related intestinal ischemia arises from patient-, device-, and procedure-related factors (4). Animal studies identify mechanical obstruction as the most direct mechanism (8–10), occurring in two clinical scenarios: malposition or migration of the balloon, so that inflation obstructs the SMA or celiac ostium; and balloon–aortic length mismatch, in which an oversized balloon or excessively long tail compresses the visceral ostia despite correct positioning. Both typically produce acute, severe ischemia that can progress rapidly to necrosis. Notably, Rastan et al. found that even with height-based sizing, 68.2% of patients have a left subclavian-to-celiac distance shorter than the balloon length—a problem amplified by spinal deformity in older patients—so overspanning of visceral arteries is common (6). Because obstruction is greatest during balloon inflation, reducing the counterpulsation ratio (from 1:1 to 1:2 or 1:3), lowering inflation pressure, or removing the catheter may temporarily relieve ischemia in high-risk patients.

Before attributing the bowel necrosis to the IABP, it is important to consider the competing and overlapping causes of bowel ischemia in this patient, because the clinical picture was consistent with multifactorial rather than single-mechanism injury. Embolic or thrombotic SMA occlusion was considered unlikely because contrast-enhanced CT demonstrated no occlusive thrombus or embolus in the mesenteric arteries and no atherosclerotic ostial stenosis. However, several well-recognized drivers of non-occlusive mesenteric ischemia (NOMI) coexisted: (1) prolonged out-of-hospital cardiac arrest with cardiopulmonary resuscitation, causing sustained systemic hypoperfusion and ischemia-reperfusion injury; (2) cardiogenic shock with a severely reduced left ventricular ejection fraction (32% on day 1); (3) therapeutic hypothermia, which reduces splanchnic blood flow and intestinal motility; and (4) high-dose vasopressor therapy, with norepinephrine escalating to 1.725 μg·kg^–1^·min^–1^ in combination with dopamine, both of which induce splanchnic vasoconstriction and are independent risk factors for NOMI. Against this background, the CTA demonstration of the balloon spanning the celiac trunk, SMA, and bilateral renal artery ostia identifies IABP-related mechanical compromise as an important additional contributor rather than the sole driver; the supporting measurements are detailed below. We therefore interpret this case as a ‘multiple-hit' injury in which balloon–aortic length mismatch likely tipped an already compromised mesenteric circulation into necrosis.

Within this multifactorial context, the mechanisms that likely acted in concert in this case of cardiogenic shock after acute myocardial infarction with IABP support are as follows: (1) Mechanical obstruction of mesenteric blood flow attributable to a mismatch between the anatomical length of the aorta and the IABP balloon. Although the 40 cc catheter was selected according to the patient's height and remained correctly positioned on radiography, the balloon was relatively oversized for this aorta: its inflated diameter (15 mm) and the minimum diameter of the descending aorta (18.5 mm) were both measured at the same level—the narrowest aortic segment within the balloon-covered region, at the level of the renal artery origins (L1)—yielding the balloon-to-aortic diameter ratio of 0.81 reported above. Diastolic balloon inflation may therefore have diminished blood flow to the visceral arteries arising from this segment (11), an effect exacerbated by pre-existing hypoperfusion, ischemia-reperfusion injury, and vasoconstrictive therapy. (2) Vasoconstriction induced by vasoactive agents: The high doses of norepinephrine and dopamine necessary to sustain hemodynamic stability can lead to visceral vasoconstriction, thereby further compromising intestinal perfusion (12). Combined with IABP, this may produce a “double-hit” effect. (3) Systemic inflammatory response: Post-cardiac arrest syndrome and ischemia-reperfusion injury drive intense systemic inflammatory responses that can impair microcirculatory blood flow, increase intestinal permeability, and lead to bacterial translocation and sepsis (13).

A further practical point concerns balloon sizing at the margins of the manufacturer's recommended range. The patient's height of 163 cm placed him essentially at the lower boundary of the 162–182 cm range specified for the 40 cc IAB-S840C catheter. Rastan et al. demonstrated that even with height-based sizing, 68.2% of patients have a left subclavian-to-celiac distance shorter than the balloon length (6); this risk is plausibly amplified at the lower end of the stated height range, where the descending aorta may be disproportionately short relative to the balloon membrane. A smaller-volume or shorter balloon (e.g., a 34 cc catheter intended by the manufacturer for patients 152–162 cm tall) might have reduced visceral artery compromise in this case, albeit at the cost of less hemodynamic support; experimental studies of short balloon catheters support this concept, showing a smaller reduction in mesenteric blood flow than with standard-size balloons while preserving circulatory efficacy (9). More broadly, height-based sizing alone may be inadequate at the margins of a device's stated range; when feasible, pre-procedural estimation of descending aortic length by CT or echocardiography, or selection of a shorter balloon, should be considered for such patients.

It should also be clarified that the balloon position measurements reported on day 0 and day 4 are not the same measurement: they used different anatomic references and modalities. The day-0 chest radiograph localized the proximal IABP marker 26.7 mm from the thoracic aortic arch—the conventional radiographic criterion for correct positioning—whereas the day-4 contrast-enhanced CT localized the distal balloon tip 32.3 mm distal to the SMA ostium, a direct vascular anatomic measurement. The radiographic position was therefore appropriate by conventional criteria, while CT revealed the balloon–aortic length mismatch that plain radiography could not detect. This discrepancy itself illustrates the limited sensitivity of chest radiography for detecting visceral artery compromise.

The classic presentation of ischemic bowel necrosis—severe abdominal pain out of proportion to the physical findings—is typically obscured in sedated and intubated IABP patients, in whom abdominal distension, gastric retention, and hematochezia become the principal clues. Diagnosis therefore requires sustained vigilance, integrating serial examination with laboratory and imaging findings. In our patient, deep sedation and targeted temperature management delayed the evaluation of progressive distension and diminishing bowel sounds; the critical warning signs were progressive abdominal distension, absent bowel sounds, and refractory shock. Notably, arterial lactate was not a reliable early warning sign: it measured 3.4 mmol/L on day 1 and had declined to 2.4 mmol/L by day 4, the day bowel necrosis was radiographically confirmed. This paradox probably reflects altered lactate kinetics in the setting of hepatic dysfunction, hypothermia, and ongoing resuscitation, and it reinforces the central message of this report: vigilance must rest on serial abdominal examination and timely imaging rather than on reassuring lactate trends, which may delay recognition of intestinal ischemia in complex critically ill patients.

CT angiography (CTA) is the most reliable imaging modality in this setting because it assesses IABP position, mesenteric vasculature, and bowel condition in a single examination. Three-dimensional reconstruction depicts the spatial relationship between the balloon and the visceral artery ostia, while bowel wall thickening, abnormal enhancement, intestinal dilation, pneumatosis intestinalis, and portal venous gas indicate ischemic necrosis; pneumatosis and portal venous gas are specific but late markers. In this case, reduced bowel wall enhancement, pneumatosis intestinalis, and hepatic portal venous gas, together with the clinical course, established the diagnosis.

The prognosis of IABP-associated ischemic bowel necrosis is dismal. Acute mesenteric ischemia itself carries a high fatality rate (14), and its coexistence with severe cardiac disease further complicates treatment. Mesenteric ischemia was the primary cause of death in 8 of 38 deceased IABP patients in the Leipzig series (6), and reported mortality among patients who develop mesenteric ischemia after cardiac surgery ranges from 70% to 100% (15).

Managing IABP-associated ischemic bowel necrosis requires a multidisciplinary approach involving cardiology, critical care, general surgery, and interventional radiology, encompassing hemodynamic stabilization, restoration of intestinal perfusion, and management of necrotic bowel. When imaging confirms malposition or balloon–aortic mismatch, repositioning, reduction of the counterpulsation ratio or inflation volume, or—if ischemic symptoms persist despite these adjustments—removal of the IABP should be considered, contingent on cardiac stability. Surgical intervention remains the definitive treatment once clinical peritonitis or imaging evidence of bowel necrosis or perforation is present. In the present case, once ischemic bowel necrosis was diagnosed on day 4, the counterpulsation ratio was reduced from 1:1 to 1:2 rather than the device being removed or repositioned, for three reasons. First, the patient was in refractory mixed cardiogenic and septic shock with an escalating vasopressor requirement (norepinephrine up to 1.725 μg·kg^–1^·min^–1^); abrupt IABP removal was judged likely to precipitate terminal circulatory collapse, whereas halving the counterpulsation frequency halved the number of inflation cycles obstructing the visceral ostia while preserving some hemodynamic support. Second, repositioning could not have helped: the problem was excessive balloon length relative to the aorta rather than catheter migration, and withdrawing the catheter far enough to clear the SMA ostium would have placed its proximal end below the left subclavian artery, abolishing effective counterpulsation. Third, CT already indicated established transmural necrosis (pneumatosis intestinalis and portal venous gas), so device manipulation alone could no longer reverse the injury; the only definitive option was laparotomy, which was not undertaken because severe, irreversible hypoxic-ischemic encephalopathy with diffuse cerebral edema was judged by the surgical team and the family to preclude meaningful recovery. The family ultimately elected to withdraw life-sustaining treatment, and the patient died. No surgical or pathological confirmation of bowel necrosis was therefore obtained; the diagnosis rests on the concordant clinical, laboratory, and CT findings described above.

This case supports six practical recommendations: (1) cautious patient selection and balloon sizing in patients at the lower end of a catheter's height range, with pre-procedural assessment of descending aortic length where available; (2) confirmation of balloon position after insertion, recognizing that chest radiography cannot exclude visceral artery compromise and that thoracoabdominal CT is preferable in high-risk patients; (3) serial abdominal examinations (distension, bowel sounds, gastric retention, bladder pressure), especially in sedated or comatose patients who cannot report pain; (4) cautious interpretation of lactate trends, which may remain falsely reassuring despite ongoing splanchnic ischemia; (5) a low threshold for early CT angiography in high-risk patients—those with post-cardiac arrest syndrome, high-dose vasopressor requirements, or unexplained abdominal findings; and (6) prompt reduction of the counterpulsation ratio or device removal at the earliest suspicion of intestinal ischemia, balanced against hemodynamic dependence and overall goals of care.

This report has several limitations. First, causality between IABP positioning and bowel necrosis cannot be proven: cardiogenic shock, prolonged cardiac arrest with resuscitation, high-dose vasopressor therapy, therapeutic hypothermia, and ischemia-reperfusion injury each independently predispose to non-occlusive mesenteric ischemia, and their relative contributions cannot be disentangled in a single case. Second, as noted above, no surgical or histopathological confirmation was obtained, and the diagnosis of transmural bowel necrosis therefore rests on radiographic criteria rather than pathology. Third, as a single case report, our findings and the recommendations derived from them are hypothesis-generating and may not be generalizable.

## Conclusion

Ischemic bowel necrosis following IABP support is a rare but highly fatal complication that usually arises from the interplay of several mechanisms rather than from device positioning alone. Even when the balloon catheter is appropriately sized based on patient height, a mismatch between aortic anatomy and balloon length may occur—particularly at the lower end of the manufacturer-specified height range—and can exacerbate splanchnic hypoperfusion caused by cardiogenic shock, prolonged resuscitation, therapeutic hypothermia, and high-dose vasopressors. Prompt thoracoabdominal CT to confirm balloon alignment, serial abdominal assessment, and a low threshold for CT angiography when abdominal signs emerge—regardless of lactate trends—are essential for early recognition of this complication.

## Data Availability

The raw data supporting the conclusions of this article will be made available by the authors, to any qualified researcher upon reasonable request.
